# Lipopolysaccharide Promotes Choroidal Neovascularization by Up-Regulation of CXCR4 and CXCR7 Expression in Choroid Endothelial Cell

**DOI:** 10.1371/journal.pone.0136175

**Published:** 2015-08-19

**Authors:** Yi-fan Feng, Hua Guo, Fei Yuan, Min-qian Shen

**Affiliations:** Department of Ophthalmology, Zhongshan Hospital, Fudan University, Shanghai, People's Republic of China; Faculty of Medicine & Health Sciences, UNITED ARAB EMIRATES

## Abstract

Stromal cell-derived factor-1 (SDF-1) has been confirmed to participate in the formation of choroidal neovascularization (CNV) via its two receptors: CXC chemokine receptors 4 (CXCR4) and CXCR7. Previous studies have indicated that the activation of Toll-like receptors (TLRs) by lipopolysaccharide (LPS) might elevate CXCR4 and/or CXCR7 expression in tumor cells, enhancing the response to SDF-1 to promote invasion and cell dissemination. However, the impact of LPS on the CXCR4 and CXCR7 expression in endothelial cells and subsequent pathological angiogenesis formation remains to be elucidated. The present study shows that LPS enhanced the CXCR4 and CXCR7 expression via activation of the TLR4 pathway in choroid-retinal endothelial (RF/6A) cells. In addition, the transcriptional regulation of CXCR4 and CXCR7 by LPS was found to be mediated by phosphorylation of the extracellular signal-related kinase (ERK) 1/2 and activation of nuclear factor kappa B (NF-κB) signaling pathways, which were blocked by ERK- or NF-κB-specific inhibitors. Furthermore, the increased CXCR4 and CXCR7 expression resulted in increased SDF-1-induced RF/6A cells proliferation, migration and tube formation. *In vivo*, LPS-treated rat had significantly higher mRNA levels of CXCR4 and CXCR7 expression and lager laser-induced CNV area than vehicle-treated rat. SDF-1 blockade with a neutralizing antibody attenuated the progression of CNV in LPS-treated rat after a single intravitreal injection. Altogether, these results demonstrated that LPS might influence CNV formation by enhancing CXCR7 and CXCR7 expression in endothelial cells, possibly providing a new perspective for the treatment of CNV-associated diseases.

## Introduction

Neovascular age-related macular degeneration (AMD), also known as wet AMD, is a leading cause of irreversible blindness in the population older than 65 years of age in the Western world [1]. Although the prevalence of wet AMD in Asians has been reported to be lower than that in Caucasians, the number of sufferers is expected to grow significantly [2]. Choroidal neovascularization (CNV), a crucial pathological aspect of wet AMD, involves angiogenesis and vasculogenesis, causing severe visual impairments due to the leakage of immature blood vessels and subsequent fibrosis [3]. Currently, vascular endothelial growth factor (VEGF) antagonists, such as bevacizumab and ranibizumab, are among the main FDA-approved drugs for the treatment of CNV; however, they require frequent intravitreal injections, and not all patients respond to such monotherapy [4]. Therefore, much research on developing novel agents targeting different molecules is needed to improve the antiangiogenic effects of CNV treatment.

Increasing evidence has suggested that inflammatory events might play a key role in the pathogenesis of AMD [5,6,7]. Previous studies have suggested that Chlamydia pneumoniae, a Gram-negative bacterium infection could be an additional risk factor for AMD progression and CNV formation [8,9,10]. Lipopolysaccharide (LPS), a component of this bacterial cell wall, is a well-known and important pathogen in pro-inflammatory responses, via binding to Toll-like receptor (TLR)-4 [11]. Research has verified that TLR4 is expressed by human retinal pigment epithelium (RPE) cells, and its physiologic and pathologic processes has been well described [12,13,14,15]. On the one hand, TLR4 was shown to mediate the recognition and clearance of effete photoreceptor outer segments (POS) by the RPE, an important function that, when impaired, can lead to AMD pathology [14]. On the other hand, over-activation of TLR4 signaling could induce the production of various pro-inflammatory cytokines, such as interleukin (IL)-6 and IL-8, which secreted by the RPE cells [13,15], and play an important role in the formation of CNV [16]. Therefore, the TLR4 pathway has attracted much attention because of its important role in the regulation of metabolic and inflammatory balance in the retina [13–15].

Stromal cell-derived factor-1 (SDF-1), also known as CXCL12, has been confirmed to play an important role in the formation of CNV via its two receptors: CXC chemokine receptors 4 (CXCR4) and CXCR7 [17,18,19,20]. Recently, a number of studies have indicated that activation of TLR4 by LPS might elevate CXCR4 and/or CXCR7 expression in tumor cells, enhancing the response to SDF-1 to promote invasion and cell dissemination [21,22]. However, much less is known about possible crosstalk between TLR4 and the CXCR4/CXCR7 axis in endothelial cell and pathological angiogenesis formation. In the current study, we demonstrated for the first time that LPS elevated the CXCR4 and CXCR7 expression subsequently increased primate choroid endothelial (RF/6A) cell angiogenesis in vitro, and laser-induced CNV development in rat eyes. Nevertheless, the size of CNV augmented by LPS-treatment was suppressed by specific inhibition of SDF-1.

## Materials and Methods

### Reagents

LPS was purchased from Sigma (St. Louis, MO, USA). Primary antibodies against CXCR4 (40kDa, ab80791), CXCR7 (42kDa, ab72100) and TLR4 (92kDa, ab30667) were purchased from Abcam Ltd. (Cambridge, UK). Rabbit anti-human antibodies against p38 MAPK (43kDa), phospho-p38 MAPK (43kDa), ERK1/2 (42/44kDa), phospho-ERK1/2 (42/44kDa), JNK (54kDa), phosphor-JNK (54kDa), NF-κB p65 (65kDa), phosphor-IKKɑ/β (85/87kDa), MEK1/2 inhibitor U0126 and JNK inhibitor SP600125 were purchased from Cell Signal Technology (Beverly, MA, USA). Rabbit polyclonal IκBɑ (41kDa), IKKɑ/β (85/87kDa) and phosphor-IκBɑ (41kDa) were purchased from Santa Cruz Biotechnology (Santa Cruz, CA, USA). HRP-conjugated monoclonal mouse anti-GAPDH antibody was purchased from Zen BioScience (Chengdu, China). Fluorescein (FITC)-conjugated goat anti-mouse IgG was purchased from Jackson ImmunoResearch Laboratories (West Grove, PA, USA). The NF-κB inhibitor BAY 11–7082 was obtained from Beyotime Institute of Biotechnology (Shanghai, China). The BCA protein assay kit and enhanced chemiluminescence (ECL) detection system were purchased from KeyGEN (Nanjing, China).

### Cell culture and animals

A choroid-retinal endothelial cell line (RF/6A) from rhesus macaques was obtained from the Cell Bank of the Chinese Academy of Sciences and was identified as being of endothelial origin by cellular morphology, growth pattern, ultrastructure, immunocytochemistry, and immunodiffusion [23]. The cells were grown at 37°C, in 5% CO_2_ and 95% humidified air in RPMI 1640 Medium (Gibco, Grand Island, NY, USA) supplemented with 10% fetal bovine serum (FBS, Gibco) and 1% penicillin/ streptomycin (HyClone, UT, USA), and the medium was changed every 2 or 3 days. Adult Male Brown Norway (BN) pigmented rats, weighing 175–200 g at 6–8 weeks of age, were purchased from SLAC Laboratory Animal Co, Ltd, Shanghai, China. All animal protocols were approved by the Ethical Committee on Animal Experiments of Animal Care Committee of Fudan University. The animals were handled in accordance with the ARVO Statement for the Use of Animals in Ophthalmic and Vision Research. Animals were allowed to acclimatize for at least 7 days before experimental manipulations.

### TLR4 gene silencing

Small interfering (si)-RNAs for TLR4 were purchased from GenenPharma (USA). The positive duplexes (siRNA-TLR4) were 5’-GGACCUCUCUCAGUGUCAATT-3’, and negative duplexes (si-Control) were 5’-UUCUCCGAACGUGUCACGUTT-3’. RF/6A cells (1×10^5^) were seeded in six-well plates and transfected with 50 nM siRNA using Lipofectamine 2000 transfection reagent (Invitrogen, Carlsbad, CA, USA), according to the manufacturers’ instructions. Briefly, 50 nM siRNA in Opti-MEM medium (Invivogen) was mixed with Lipofectamine 2000 and incubated for 25 min at room temperature before adding the mixture to the cells. After 48 h, the level of silencing was determined by real-time PCR and western blotting.

### Immunofluorescence staining

RF/6A cells were seeded in a 24-well dish (3 × 10^4^ cells/well) and cultured for 24 h. The cells were then stimulated with LPS (1 μg/ml) for 24 h and were fixed with 4% paraformaldehyde for 20 minutes at room temperature. After rinsing with phosphate-buffered solution (PBS), the cells were blocked with 5% horse serum in PBS for 1 hour at room temperature and then were incubated with TLR4 antibody (1:50 dilution) overnight at 4°C. On the following day, the cells were incubated with the secondary antibody (1:200 dilution) for 2 h at room temperature, washed three times with PBS, and incubated with 100 ng/mL DAPI for 5 mins. All of the images were captured with a fluorescence microscope (BX50 Olympus, Tokyo, Japan).

### Cloning of the CXCR4 and CXCR7 promoters

The promoter region of the CXCR4 and CXCR7 genes, from -2,000 bp to +50 bp, relative to the transcription start site were amplified according to previous report [24]. The confirmed sequence was then inserted into a pGL3-Basic vector (Promega). The pGL3-Basic vector was digested by KpnI and XhoI (or XhoI /HindIII for CXCR7; all restriction enzymes were acquired from MBI Fermentas), and the amplified CXCR4 and CXCR7 promoter fragments were inserted through ligation. The cloned pGL3-CXCR4 and pGL3-CXCR7 constructs were confirmed by sequencing. The sequentially shorter CXCR4 and CXCR7 promoter fragments were amplified by standard PCR methods, sequenced, and cloned to the pGL3 vector.

### Luciferase reporter gene assays

For the CXCR4 and CXCR7 reporter assay, RF/6A cells were co-transfected with a mixture of 1 μg of CXCR4 or CXCR7 luciferase reporter plasmid and 0.1 μg of constitutive Renilla luciferase plasmid (Promega Corporation, Madison, WI, USA) using Lipofectamine 2000. For the NF-κB p65 reporter assay, RF/6A cells were co-transfected with a mixture of 1 μg of NF-κB luciferase reporter plasmid and 0.1 μg of Renilla luciferase plasmid. LPS (μg/ml), with or without BAY 11–7082, was added to the medium 6 h after transfection. The luciferase activity in the cells was measured with a Dual-Luciferase Reporter Assay system, according to the manufacturer’s instructions (Promega). Relative luciferase activities were expressed as the ratio of firefly to Renilla luciferase activity.

### Western blotting

Total cellular and nuclear proteins were extracted according to the instructions of the cytoplasmic and nuclear protein extraction Kit (Beyotime). The nuclear extracts were used to determine NF-κB p65 protein levels and the cytoplasmic extracts were used to determine IκB and IKK levels. The protein concentration was measured using a BCA assay kit, according to the manufacturer’s instruction. Forty micrograms of protein were separated on 10% SDS-PAGE and transferred onto PVDF membranes (Millipore, Bedford, MA, USA). After the blot was incubated in blocking solution (5% skim milk in PBS, 0.1% Tween-20), the membranes were incubated overnight with primary antibodies at 4°C. The membrane was then washed with TBST three times, incubated with HRP-conjugated secondary antibody for 2 hours at room temperature, and detected with ECL detection reagents, according to the manufacturers’ recommendations.

### Quantitative real-time PCR

Total RNA from RF/6A or the posterior part of the eyeball (the retina, RPE and choroid) at 24 h after laser treatment was isolated using Trizol (Invitrogen, Carlsbad, CA, USA) and then was reverse transcribed with PrimeScript RT Master mix (Takara, Otsu, Japan). Quantitative real-time polymerase chain reaction (qRT-PCR) was conducted using an Eppendorf Mastercycler ep realplex machine (Eppendorf, Germany) and a SYBR Premix Kit (Takara), according to the manufacturers’ instructions. The sequences of the specific primers were presented in Table 1. Relative mRNA expression levels were calculated by the 2^-△△Ct^ method. GAPDH was used as a reference gene.

**Table 1:** Primer pairs used for analysis.

*Species*	*Gene*	*Forward (5’-3’)*	*Reverse (5’-3’)*
Macaca mulatta	GAPDH	ctttggtatcgtggaaggactc	gtagaggcagggatgttct
Macaca mulatta	CXCR4	atcctcatcctggctttcttc	caaactcacacccttgcttgat
Macaca mulatta	CXCR7	tgccagacacctactacctgaa	gaagcccaagaccacagagac
Macaca mulatta	TLR4	tgtcccagcacttcatccag	gggtcttctccaccttctgc
Rattus norvegicus	GAPDH	gatgacccagatcatgtttga	gagagcatagccctcgtag
Rattus norvegicus	CXCR4	tgcagcaggtagcagtgaaa	tgtatatactcacactgatcggttc
Rattus norvegicus	CXCR7	ggacaccccacaaatcactct	ggagccttccgttcttaggg

GAPDH = glyceraldehyde-3-phosphate dehydrogenase; CXCR4/7 = chemokine (C-X-C motif) receptor 4/7; TLR4 = toll like receptor 4.

### Proliferation assay

Cell Counting Kit-8 (CCK8, KeyGEN) was applied according to the manufacturer’s instructions. Twenty thousand RF/6A cells per well were placed on a 96-well plate, in 100 μl of 1% FBS medium without (control) or with the addition of CXCL12 (100 ng/ml). In some of the experiments, the RF/6A cells were pretreated with LPS (1 μg/ml, 24 h) alone or in combination with NF-κB p65 inhibitor of BAY 11–7082 (10 μM) for 12 h. At 24 h, 48 h and 72 h after SDF-1 treatment, CCK-8 reagent was added to the cells 2 h before measuring the absorbance value at 490 nm using a microplate reader, according to the manufacturer’s instructions.

### Migration assay

Cell migration assay was performed on a 24-well plate with 8.0-μm pore-size transwell inserts (Nunc, Roskilde, Denmark). Twenty thousand RF/6A cells were pretreated with LPS (1 μg/ml), alone or in combination with BAY 11–7082 (10 μM), and then were seeded in the upper chamber with 200 μl of RPMI 1640 medium supplemented with 0.5% FBS. To this mixture, 600 μl of medium containing 0.5% FBS, without (control) or with recombinant human SDF-1 (100 ng/ml, PeproTech), was added to the bottom chamber. After incubation at 37°C for 24 h, the inserts were fixed with 4% paraformaldehyde, and the cells were stained with Giemsa solution. Cell numbers from 5 random visual fields were counted under a microscope.

### Tube formation assay

The tube formation assay was conducted as previously described. Briefly, aliquots (150 μl) of Matrigel (BD Biosciences) were added to a 48-well plate and were incubated at 37°C for 30 min. The cells were resuspended in supernatants collected from each pretreatment and then were seeded onto the gel (2 × 10 ^4^ cells/well); 100 ng/ml SDF-1 was added if needed. Five random fields from each well were chosen and photographed after 8 h. Networks of tube-like structures were measured using Image Pro Plus software, version 6.0 (MediaCybernetic, Silver Spring, MA, USA).

### Vitreous injection

At 1 day before laser irradiation, 10 μl LPS (10 ng/ml) was injected into the vitreous cavity from the temporal limbus of the eye by using a 32-gauge needle (Hamilton, Reno, NV) and the dose was based on previous studies in mice [8]. Because the total amount of ocular fluid was approximately 55 μl [25], the final concentration of LPS in the eye was approximately 2 ng/ml. In some eyes, immediately after CNV induction, 1 μg anti-SDF-1 antibody (MAB310; R&D Systems) was injected intravitreally from the temporal limbus of the same eye in a 10-μl volume, according to.a previous study by Otsuka et al. [26]. The control rats received the same volume of PBS 1 days before laser irradiation.

### Laser-induced CNV model

CNV was induced by photocoagulation and was evaluated as previous described [27]. In brief, rats were anaesthetized with 2% sodium pentobarbital (50 mg/kg, Sigma), and pupils of both eyes were dilated with 1% tropicamide (Alcon Laboratories, Inc, Fort Worth, TX, USA). Laser photocoagulation (532 nm wavelength, 100 mW power, 75 μm spot size, 100 ms duration) was performed bilaterally in each rat. Four to six laser spots were applied around the optic nerve using a slit lamp delivery system and using a cover slip as a contact lens. All laser burns had the appearance of a cavitation bubble. Spots containing haemorrhage or failing to develop a bubble at the laser site were excluded from the analysis.

### Fundus fluorescein angiography

The rats were anesthetized, and 10% fluorescein sodium (0.1 ml/kg, Fluorescite; Alcon, Fort Worth, TX) was injected by peritoneal injection. Consecutive recording of the ocular fundus was performed by a commercial camera and imaging system (TRC-50DX and IMAGEnet ver. 1.53; Topcon, Tokyo, Japan) with a 20-D lens in contact with the fundus camera lens at baseline and 1 week after laser photocoagulation.

### Choroidal flatmount

One week after laser photocoagulation, rats were killed and the eyes were processed for fluorescent-labeled isolectin stain of CNV lesions according to previous reports [28]. Briefly, after fixation with 4% paraformaldehyde and incubation with a blocking solution containing 0.5% BSA and 0.1% TritonX-100 in PBS, the eyecups were stained with 1:100 diluted FITC-conjugated BS-I isolectin B4 (IB4 lectin, Sigma, St. Louis, MO) in the blocking buffer at 4°C overnight. With four to approximately six relaxing radial cuts, RPE-choroid-sclera complex was mounted flat on a glass slide with the RPE side upward. The area of CNV lesion in each sample was measured using imaging software (SPOT Advanced; Diagnostic Instruments, Inc., Sterling Heights, MI, USA).

### ELISA assay

The concentrations of IL-6, IL-10 and SDF-1 in the intraocular fluid (mixture of aqueous humor and vitreous fluid) were measured as previous described [8]. Twenty-four hours after LPS inoculation and photocoagulation, eyes were enucleated under deep anesthesia, the conjunctival tissue was removed, and the remaining eye tissues (cornea, iris, vitreous body, retina, choroids, and sclera) were homogenized (Biomasher; Nippi Inc., Tokyo, Japan). After centrifugation at 12,000g for 30 minutes, supernatants were collected, and the concentrations of cytokine were measured using ELISA kits (R&D Systems, Inc., Minneapolis, M N, USA).

### Statistical Analysis

Student’s *t* test and one-way ANOVA were implemented for all of the statistical data. All of the analyses were performed using GraphPad Prism software (Graphpad Software, La Jolla, CA, USA). Values are expressed as the means ± SDs, and statistical significance was set at P < 0.05.

## Results

### LPS up-regulates CXCR4 and CXCR7 expression in time- and dose-dependent manners

**Fig 1:**
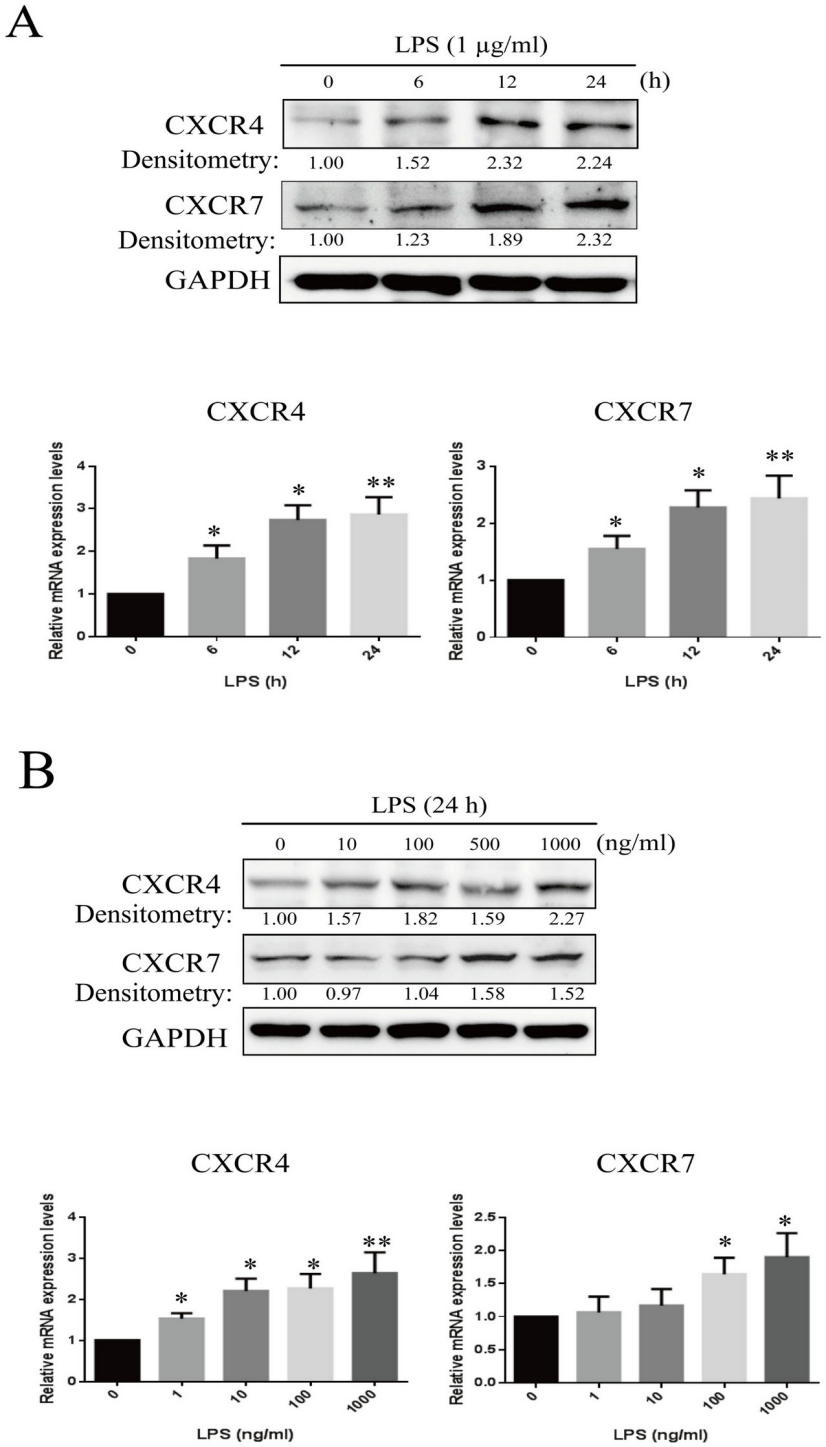
Effects of LPS on CXCR4 and CXCR7 expression in RF/6A cells. Expression levels of CXCR4 and CXCR7 were measured by real-time PCR and western blots. (**A**) The mRNA and protein levels of CXCR4 and CXCR7 in RF/6A cells under LPS exposure (1 μ/ml) at different time points. (**B**) The mRNA and protein levels of CXCR4 and CXCR7 in RF/6A cells under different doses of LPS exposure for 24 h. Data are shown as the mean ± SD of four separate experiments. Statistical significance determined using one-way ANOVA with *P < 0.05, **P < 0.01.

**Fig 2:**
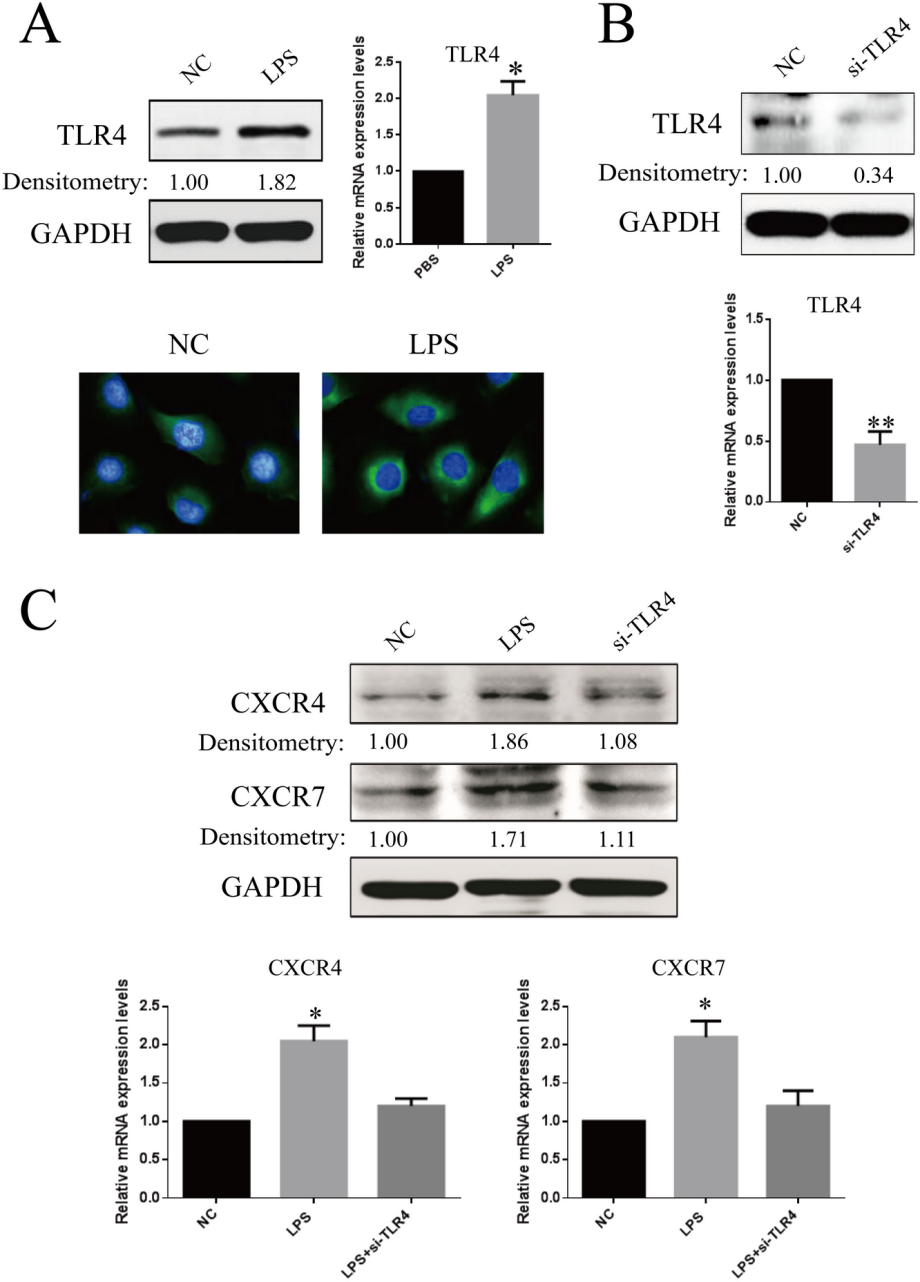
Confirmation of TLR4 expression in RF/6A cells and the effects of TLR4 knockdown on LPS-induced CXCR4 and CXCR7 expression. (**A**) RF/6A cells were stimulated by LPS (1μ/ml) and were compared for the expression of TLR4 with that of untreated cells. After 24 h, the amounts of TLR4 mRNA and protein were quantified by real-time RT-PCR and western blot. In addition, immunofluorescence microscopic analysis was performed for the identification of TLR4 location and expression. TLR4 was labeled by green fluorescence (FITC), and the nuclei were counterstained with DAPI (blue). (**B**) RF/6A cells transfected with the TLR4 siRNA sequence exhibited a marked reduction in TLR4 mRNA and protein levels, compared with negative control sequence (control). (**C**) After LPS treatment, western blot and real-time RT-PCR analyses shown that knockdown of TLR4 inhibited LPS-mediated CXCR4 and CXCR7 expression. Data are shown as the mean ± SD of three separate experiments. Statistical significance determined using Student’s *t* test or one-way ANOVA with *P < 0.05, **P < 0.01.

To examine the effects of LPS on CXCR4 and CXCR7 expression, RF/6A cells were treated with different concentrations (0–1000 ng/ml) of LPS in serum-free medium for different time periods (0–24 h). Western blot results showed that LPS (1 ug/ml) enhanced the protein expression of both CXCR4 and CXCR7 in a time-dependent manner, peaking at 12–24 h **(Fig 1A)**. Densitometric analysis showed a significant increase (> 2.0-fold; P < 0.01) in the expression of both CXCR4 and CXCR7 in the LPS-treated compared with untreated RF/6A. LPS also enhanced the expression levels of CXCR4 and CXCR7 in a dose-dependent manner, with a peak effect at 1 μg/ml (> 2.0-fold; P < 0.01) **(Fig 2B)**. The changes of mRNA levels detected by qRT-PCR were consistent with the protein expression.

### Knockdown of TLR4 inhibits LPS-mediated CXCR4 and CXCR7 expression

To investigate the role of TLR4 in LPS-mediated CXCR4 and CXCR7 expression, we first confirmed TLR4 expression on RF/6A cells. The results of western blot, qRT-PCR and immunostaining showed that the expression of TLR4 on unstimulated RF/6A cells was not very high, but the expression was augmented (1.82-fold, P < 0.05) by LPS stimulation **(Fig 2A)**. Then, we subjected RF/6A cells expressing TLR4 to transient transfection with the siRNA specific for the TLR4 gene. Cells transfected with the TLR4 siRNA sequence showed a significant reduction in the TLR4 mRNA and protein levels (P < 0.01), compared with those of cells transfected with negative control sequence **(Fig 2B)**. Furthermore, incubation of RF/6A cells transfected with the TLR4 siRNA sequence with LPS did not reveal an increase in the expression of CXCR4 and CXCR7 mRNA and protein. Conversely, transfection with the negative control sequence in cells resulted in a significant increase in CXCR4 and CXCR7 expression in response to LPS **(Fig 2C)**. These results suggested that LPS could up-regulate the expressions of both CXCR4 and CXCR7 via binding to TLR4.

### Involvement of the ERK and NF-ΚB signaling pathways in LPS-mediated up-regulation of CXCR4 and CXCR7

Because LPS activates several signaling pathways, including NF-κB and MAPK (e.g. ERK1/2, JNK and p-38) [20], we performed western blot analysis to elucidate the signal-transduction mechanisms involved in the LPS-induced up-regulation of CXCR4 and CXCR7. As shown in **S1A Fig**, LPS activated ERK1/2 and JNK in a time-dependent manner, as evidenced by the increases in phosphorylated ERK1/2 and JNK, but not p-38. Inhibitors of ERK1/2 (U0126, 10 μM) and JNK (SP600125, 10 μM) prevented the LPS-induced phosphorylation of ERK1/2 and JNK **(S1B Fig)**. Additionally, stimulation of cells with LPS induced IKKɑ/β, IκBɑ phosphorylation and IκBɑ degradation in the cytoplasm and NF-κB p65 up-regulation in the nucleus **(S1C Fig)**. Using fluorescence microscopy, we demonstrated translocation of NF-κB from the cytosol to the nucleus, whereas NF-κB p65 translocation was efficiently inhibited by BAY 11–7082 (10 μM) pretreatment **(S1D Fig)**. In addition, transient transfection of the NF-κB p65 promoter luciferase construct, followed by incubation with LPS for 24 h, led to an increase in NF-κB p65 promoter activity in RF/6A cells **(S1E Fig)**.

**Fig 3:**
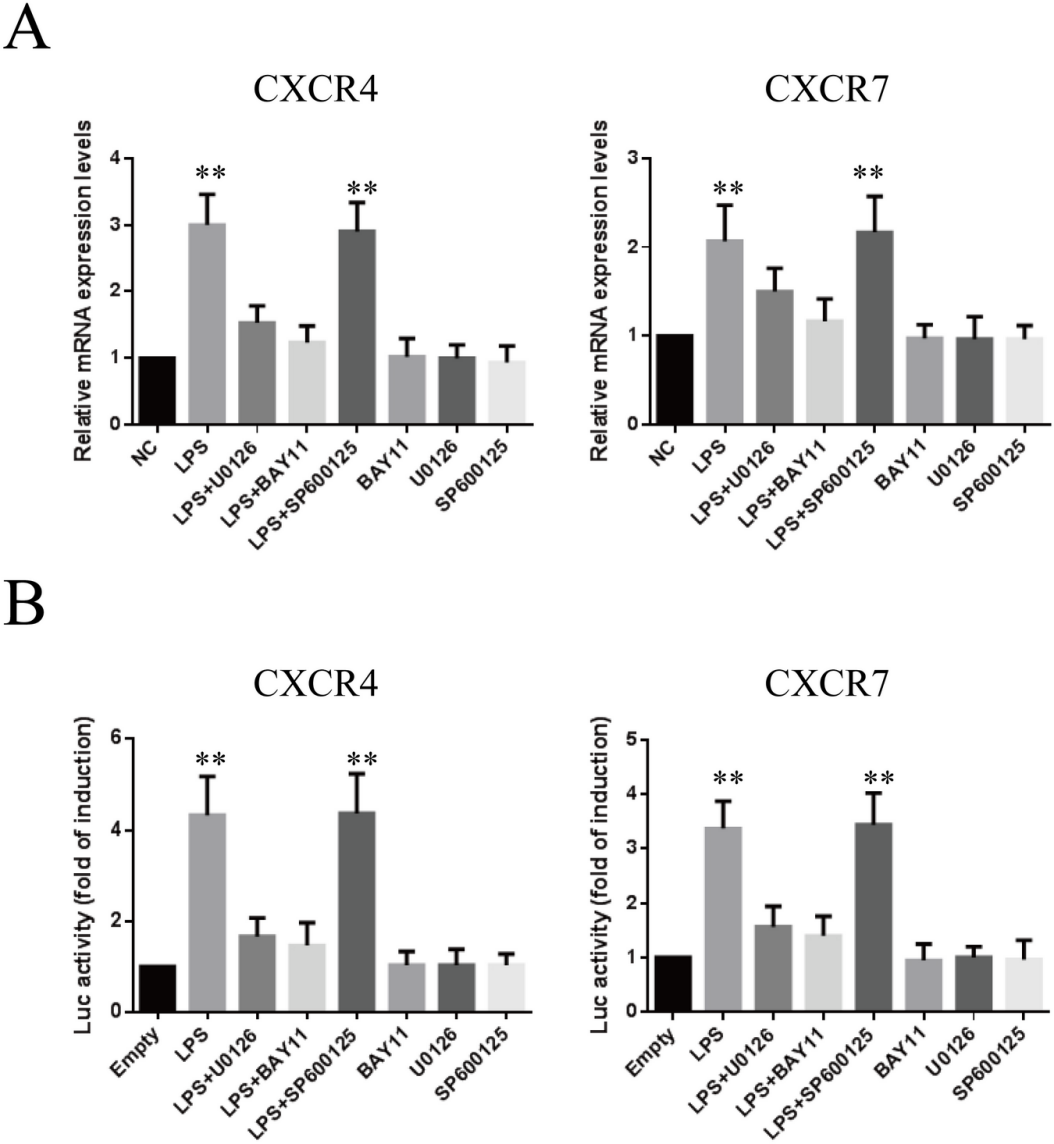
Effects of different inhibitors on LPS-induced CXCR4 and CXCR7 mRNA expression and promoter activity. **(A)** Cells were pretreated with inhibitors specific for JNK (SP600125), ERK1/2 (U0126), and NF-κB (BAY 11–7082) at 10 μM concentrations for 1 h and then with LPS (1 μg/ml) for 24 h; CXCR4 and CXCR7 mRNA levels were determined by real-time PCR. (B) Luciferase activity of CXCR4 and CXCR7 promoter constructs transiently transfected into RF/6A cells and then pretreated with SP600125 (10 μM), U0126 (10 μM) or BAY 11–7082 (10 μM) for 1 h, followed by treatment with or without LPS (1 μg/ml) for an additional 24 h. The results are expressed as fold changes of luciferase activity (relative light units, RLUs), compared to the group without LPS treatment (**P < 0.01). Data are mean ± SD for four independent experiments.

We then examined whether the ERK1/2, JNK and NF-κB p65 pathways were related to the increase in CXCR4 and CXCR7 expression induced by LPS. As shown in **Fig 3A**, LPS-induced increases in CXCR4 and CXCR7 mRNA expression were markedly antagonized by treatment with the ERK1/2 inhibitor U0126 (10 μM) and the NF-κB inhibitor BAY 11–7082 (10 μM), but not by the JNK inhibitor SP600125 (10 μM). To confirm these results, we tested the effects of LPS on CXCR4 and CXCR7 transcription. Similar to the results with PCR, expression of the CXCR4 and CXCR7 reporter gene was enhanced by LPS stimulation and was specifically abolished by U0126 or BAY 11–7082 pretreatment **(Fig 3B)**.

### SDF-1-induced proliferation, migration and tube formation are enhanced by LPS pretreatment

**Fig 4:**
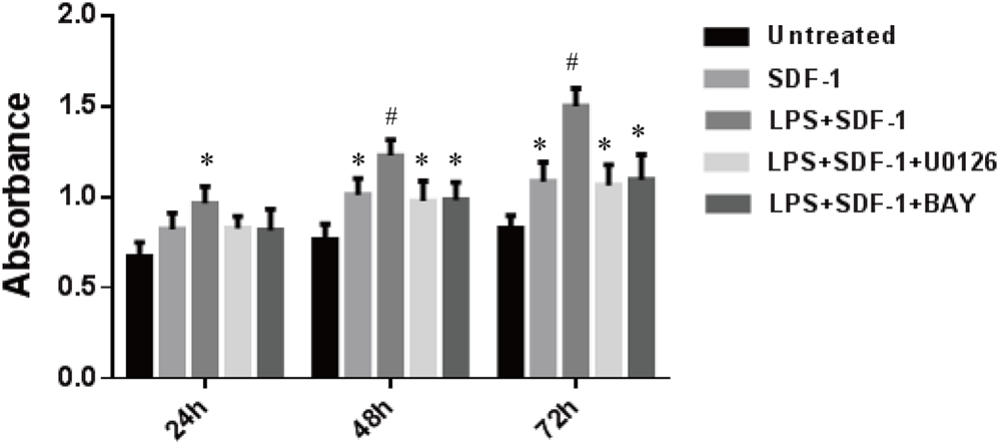
Effects of LPS on the SDF-1-induced proliferation of RF/6A cells. Cell proliferation was measured by CCK-8 at 24, 48 and 72 h. RF/6A cells pretreated with LPS (1 μg/ml) for 24 h proliferated significantly in response to SDF-1 (100 ng/ml), but this effect was attenuated by treatment with U0126 or BAY 11–7082. Data are mean ± SD for three independent experiments. Statistical significance determined using one-way ANOVA with *P < 0.05, **P < 0.01.

**Fig 5:**
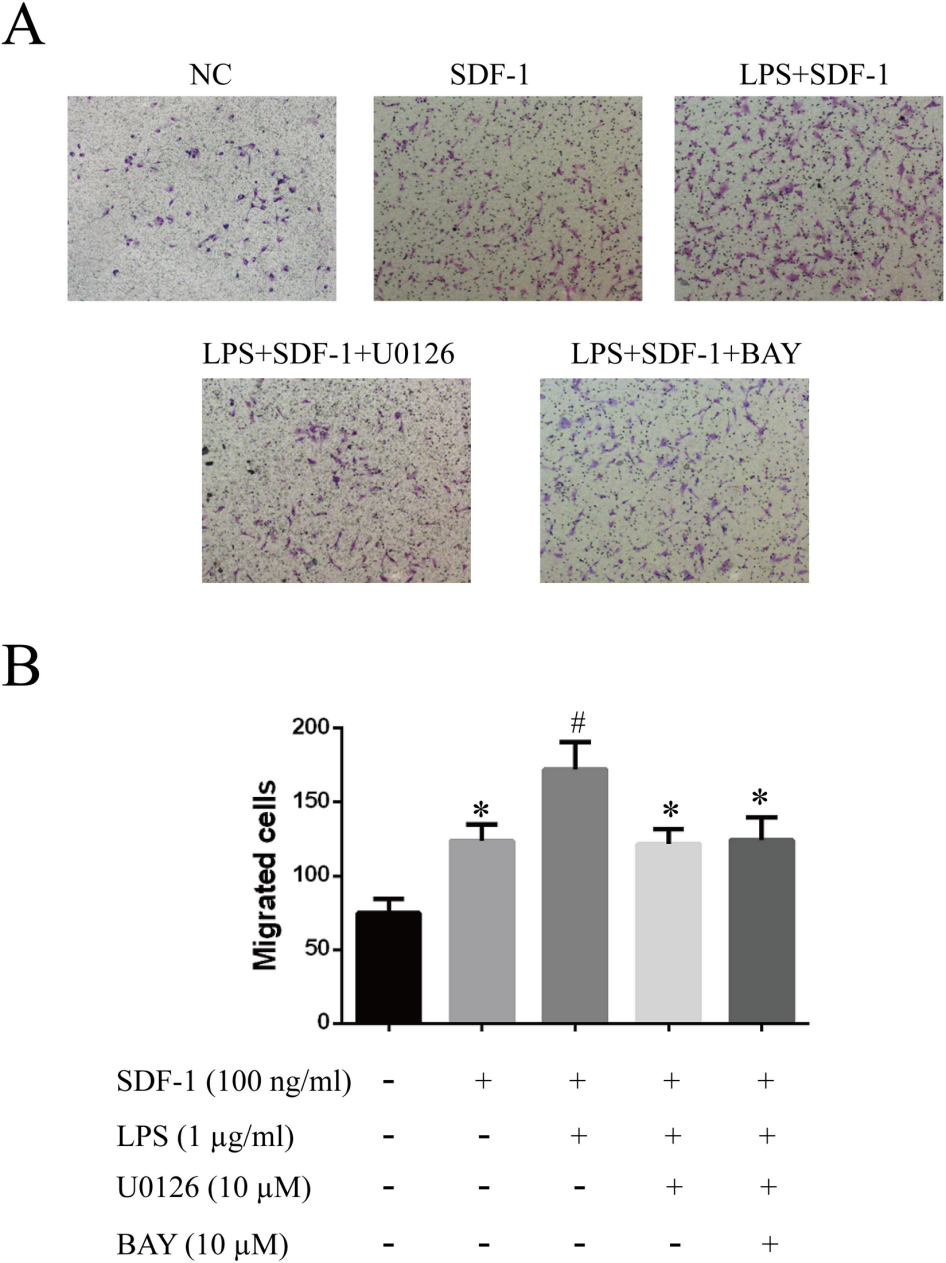
Effects of LPS on the SDF-1-induced migration of RF/6A cells. The migratory activity of RF/6A cells after different treatments was estimated based on the number of cells that migrated through the filter inserts. Representative images of cell migration are given: untreated (**A**); SDF-1 (100 ng/ml) alone in the lower chamber (**B**); LPS (1 μg/ml) pretreatment plus SDF-1 (100 ng/ml) in the lower chamber (**C**); combined LPS and U0126 pretreatment plus SDF-1 in the lower chamber (**D**); combined LPS and BAY 11–7082 pretreatment plus SDF-1 in the lower chamber (**E**). (**F**) Quantitative analysis of the number of migrated cells, expressed as the mean ± SD. Statistical significance determined using one-way ANOVA with *P < 0.05, **P < 0.01.

**Fig 6:**
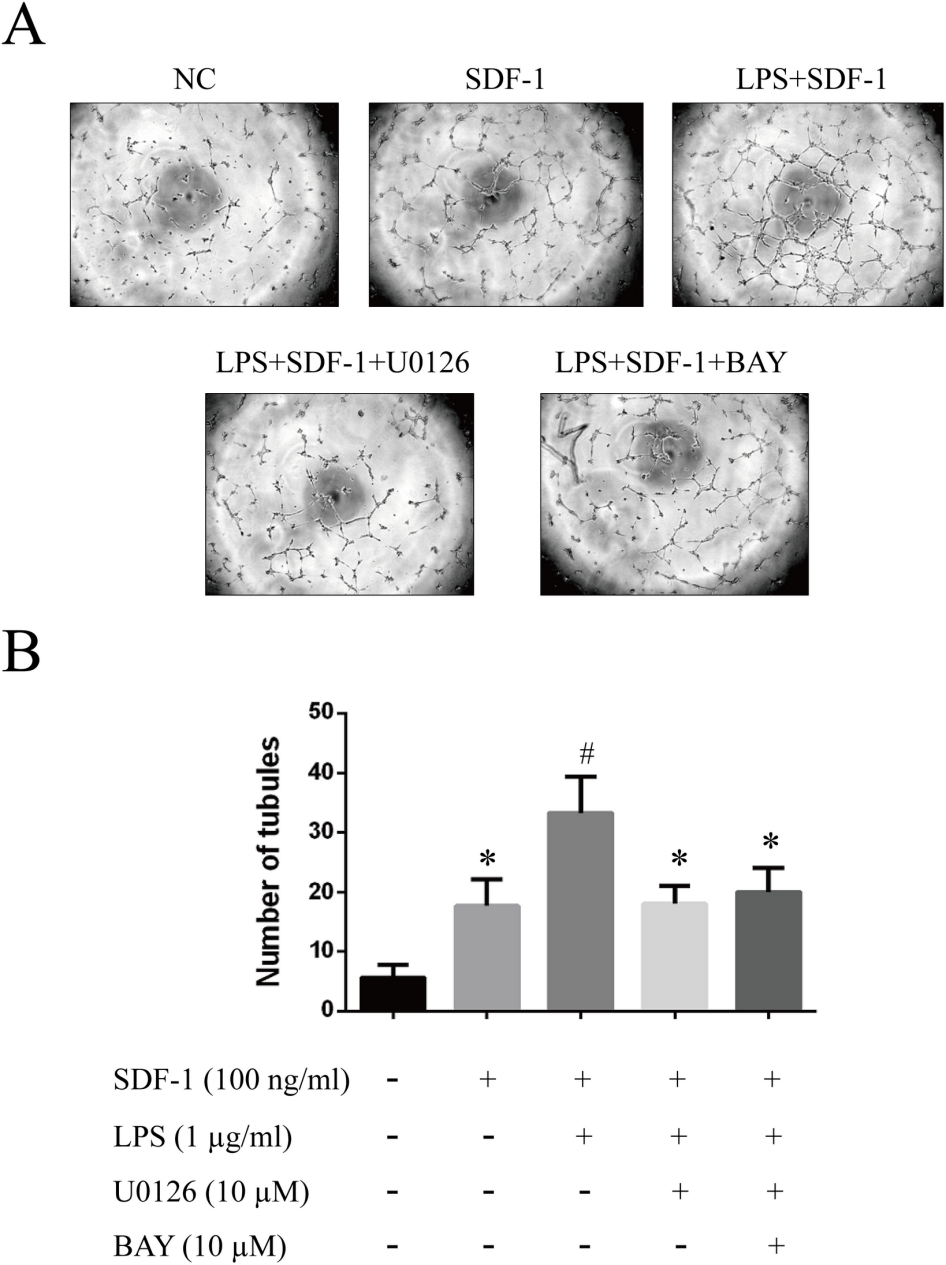
Effects of LPS on SDF-1-induced RF/6A cell tube formation. Networks of tube-like structures were measured in a blinded manner. Representative images of tube-like structures are given: untreated (**A**); SDF-1 (100 ng/ml) alone in the lower chamber (**B**); LPS (1 μg/ml) pretreatment plus CXCL12 (100 ng/ml) in the lower chamber (**C**); combined LPS and U0126 pretreatment plus SDF-1 in the lower chamber (**D**); combined LPS and BAY 11–7082 pretreatment plus SDF-1 in the lower chamber (**E**). (**F**) Quantitative analysis of the number of tubules, expressed as the mean ± SD. Statistical significance determined using one-way ANOVA with *P < 0.05, **P < 0.01.

To investigate whether increased CXCR4 and CXCR7 expression enhanced the SDF-1-induced proliferation, migration and tube formation of RF/6A cells, we pretreated the cells with 1 μg/ml LPS, with or without U0126 and BAY 11–7082, for 24 h, followed by treatment with SDF-1 (100 ng/ml). The results, shown in **Figs 4, 5 and 6**, indicated that SDF-1 promoted RF/6A cell proliferation (OD_72h_: 1.23 ± 0.12 vs. 0.86 ± 0.07, P < 0.05), migration (129.0 ± 16.1 vs. 71.2 ± 11.4, P < 0.05) and tube formation (17.3 ± 4.1 vs. 6.5 ± 1.8, P < 0.05) when compared to control. These effects were significantly enhanced by LPS pretreatment (OD_72h_: 1.54 ± 0.13 vs. 1.23 ± 0.12, P < 0.05; 168.8 ± 20.1 vs. 129.0 ± 16.1, P < 0.05; 33.8 ± 6.9 vs. 17.3 ± 4.1, P < 0.05), but were reversed by treatment with MEK or NF-κB inhibitors. Together, these results indicated that the up-regulation of CXCR4 and CXCR7 induced by LPS sensitized RF/6A cells to respond to SDF-1, mediating the cells’ proliferation, migration and tube formation.

### Vitreal SDF-1 Neutralization suppressed experimental CNV in LPS-treated rat

To investigate CXCR4 and CXCR7 expression in the choroid, 3 days after laser-induced CNV in BN rats, RPE-choroid complexes was harvested and expression of these proteins was analyzed by qRT-PCR. The mRNA level of CXCR4 and CXCR7 in the LPS-pretreated rat was substantially higher (> 1.5-fold, P < 0.05), 3 days after RD, compared with the PBS-inoculated rat **(S2A Fig)**. We also measured intraocular cytokine concentrations in LPS-inoculated eyes. Three days after LPS inoculation and photocoagulation, eyes were enucleated and their intraocular fluid (mixture of aqueous humor and vitreous fluid) were obtained for ELISA. The concentrations of SDF-1 and IL-6 in PBS-inoculated mice were 32.49 ± 7.12 pg/ml and 16.36 ± 4.55 pg/ml. In contrast, both cytokines were significantly increased in LPS-inoculated rat (SDF-1, 52.06 ± 22.67 pg/ml [P < 0.05]; IL-6, 65.48 ± 17.19 pg/ml [P < 0.01]). However, the concentration of IL-10 was not significantly different between the two groups **(S2B Fig)**.

**Fig 7:**
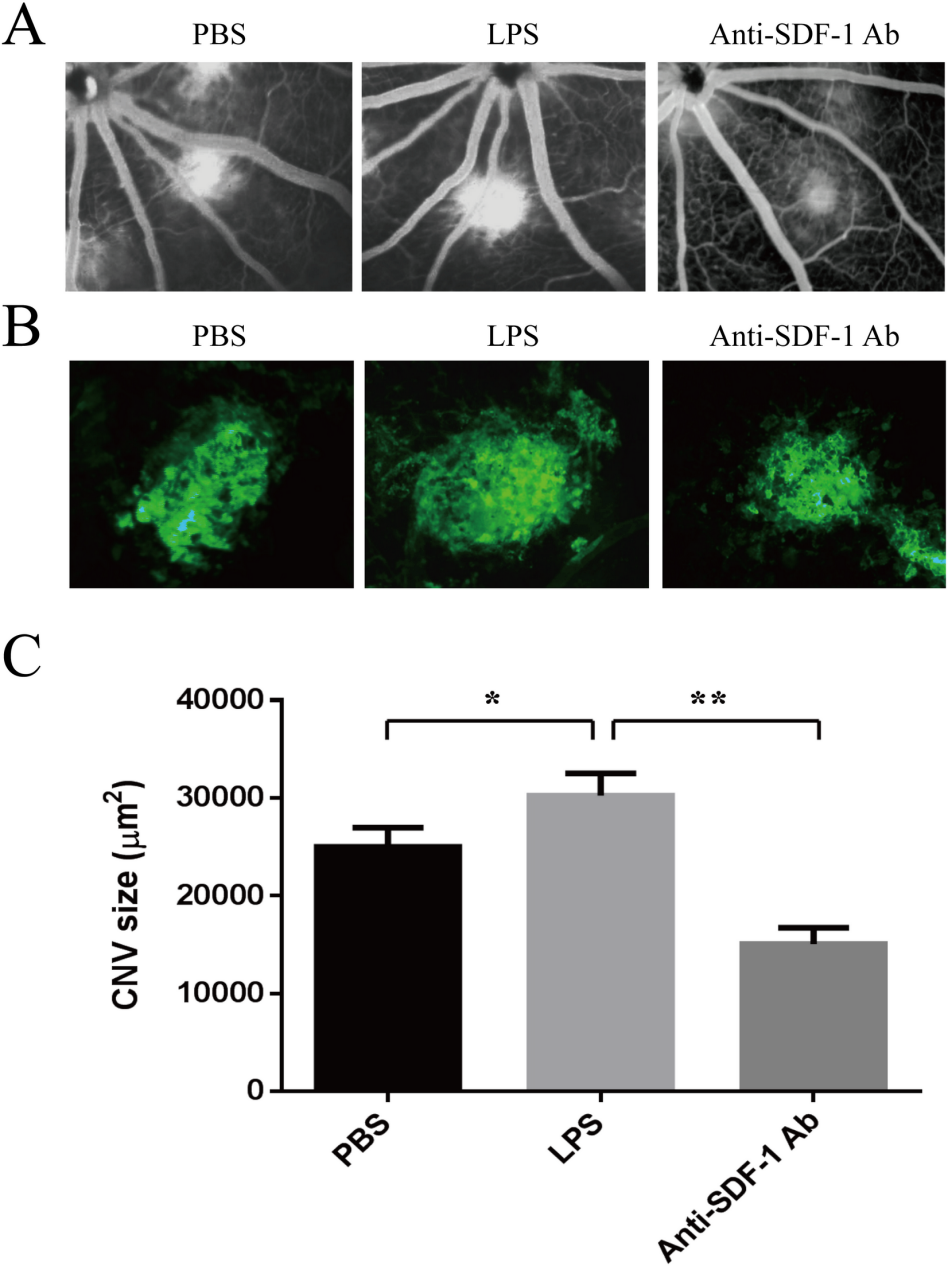
Effect of SDF-1 Neutralization on laser-induced CNV in LPS-treated mice. Male adult Brown Norway rats received a single intravitreal injection of 10 μl LPS at 10 ng/ml (equivalent to an intravitreal centration of approximately 2 ng/ml) in eyes at one day before laser photocoagulation. The control rats received the same volume of vehicle (PBS). For some of LPS-treated rats, 1 μg anti-SDF-1 antibody in a 10-μl volume was injected immediately after laser photocoagulation. (A) Representative fluorescein angiography at 7 days after laser photocoagulation. (B) Representative images of isolectin B4 stain of RPE-choroid-sclera whole mount that performed 7 days after laser. (C) Quantitative measurement of CNV area. Data shown are mean ± SD of triplicate samples and are representative of four independent experiments. Statistical significance determined using one-way ANOVA with *P < 0.05, **P < 0.01.

In order to confirm the assumption that LPS increase size of CNV via regulating the SDF-1/CXCR4/CXCR7 axis in vivo, we used a laser-induced CNV model. LPS was inoculated into the vitreous cavity of BN rats at 1 day before of laser treatment, and some of them were intravitreally injected with anti-SDF-1 antibody in the same eyes immediately after CNV induction. The appearance of CNV in choroidal flat mounts was visualized by fluorescence angiography on day 7. Fluorescein angiography revealed LPS markedly increase CNV leakage compared with PBS-inoculated group, but this effect was reversed by anti-SDF-1 antibody **(Fig 7A and 7B)**. The size of CNV lesion was also significantly larger in LPS-treated group (3109.27 μm^2^) than that in the PBS-treated group (2492.56 μm^2^). The mean value of CNV size was significantly reduced to nearly half of the value in the LPS–treated group by anti-SDF-1 antibody (1513.56 μm^2^, P < 0.01) **(Fig 7C)**.

## Discussion

Though the exact mechanisms that induce AMD/CNV has not been fully understood, chronic inflammation is identified as a candidate etiology and at least one of the essential processes [5,6,7]. TLR4, which is known to the specific receptor of LPS, plays a pivotal role in the innate immunity of the eye. A number of studies have shown that oxidative stress, mitochondrial DNA damage, chronic inflammation, and angiogenesis (basic pathologic features of AMD) in the choroid (particularly in the RPE cells) can be induced by the TLR4-mediated pathway [13,14]. However, until now, the role of the TLR4 pathway has not been studied in regional cells, such as choroidal endothelial cells. In the present study, we demonstrated that LPS elevated CXCR4 and CXCR7 expression in RF/6A cells via TLR4, and this function required the ERK1/2 and NF-κB signaling pathways. Moreover, the up-regulation of CXCR4 and CXCR7 expression increased SDF-1-induced proliferation, migration and tube formation. Furthermore, in vivo study confirmed the “crosstalk” between LPS and SDF-1/CXCR4/CXCR7 axis in laser-induced CNV. Our findings provided a novel molecular basis for the angiogenesis observed during CNV formation.

Studies in animal models have suggested that SDF-1 might be an important factor in the differentiation of bone marrow-derived cells (BMCs) into endothelial cells and that it might participate in CNV [29]. CXCR4 was the first receptor identified as binding to SDF-1, and their partnership had long been thought to be uniquely selective until the recent discovery of CXCR7, a new receptor for SDF-1 that also binds to the I-TAC chemokine [30]. In our previous work, we confirmed that CXCR4, but not CXCR7, mediated SDF-1-induced RF/6A cell proliferation and migration, while CXCR7 was essential for SDF-1-induced resistance to apoptosis; both CXCR4 and CXCR7 were crucial in tube formation [31]. These findings implicated the SDF-1/CXCR4/CXCR7 axis in CNV formation.

The expression of CXCR4 and CXCR7 can be regulated by many cytokines, such as hypoxia-inducible factor-1 (HIF-1) [31,32,33] and transforming growth factor beta (TGF-β) [17,34]. Recently, increasing evidence has suggested that alteration of CXCR4 or CXCR7 expression mediated by LPS-TLR4 enhances the invasiveness of tumor cells [21,22]. Takabayashi et al. [22] reported that LPS treatment increased the mRNA expression of CXCR4 and promoted the invasiveness in human oral carcinoma T3M-1 cells. More recently, Xu et al. [21] found that exposure to LPS could elevate CXCR7 but not CXCR4 expression in human colorectal carcinoma SW480 and Colo 205 cell lines. In our present work, LPS treatment up-regulated the mRNA and protein expression of both CXCR4 and CXCR7, subsequently enhanced the RF/6A cells’ proliferation, migration and tube formation. Knockdown of TLR4 inhibited LPS-mediated CXCR4 and CXCR7 expression alterations.

To explore further the possible molecular mechanism involved in LPS-induced transcriptional up-regulation of CXCR4 and CXCR7, we investigated the effects of LPS on the involved signaling transduction pathways, such as MAPK and NF-κB signaling. To the best of our knowledge, this study was the first demonstrating that the NF-κB and ERK1/2 signaling pathways were involved in LPS-induced CXCR4 and CXCR7 expression in RF/6A cells. We noted that NF-κB activation was dramatically induced by LPS stimulation of RF/6A cells, through IκBɑ degradation and phosphorylation of IκBɑ, IKKɑ and p65, a subunit of NF-κB. In addition to the NF-κB pathway, LPS also triggered MAPK signaling pathways, including ERK1/2 and JNK. The results of qRT- PCR showed that the up-regulation of CXCR4 and CXCR7 mRNA expression was markedly antagonized by the ERK1/2 inhibitor U0126 and the NF-κB inhibitor BAY 11–7082, but not by the JNK inhibitor SP600125. Our results were partly different from a previous study, which showed that specific inhibitor of JNK clearly repressed the expression of SDF-1, CXCR4 induced by LPS in human dental pulp stem cells [35]. These conflicting data indicate that the signal pathways of controlling CXCR4 and CXCR7 expression activated by LPS depending on the cell type or tissue origin. Because our results showed that the regulation of CXCR4 and CXCR7 occurred mainly at the mRNA level, we further investigated the transcriptional regulation of LPS-induced CXCR4 and CXCR7 expression. Similarly, our results demonstrated that the NF-κB and ERK1/2 signaling pathways were both involved in the regulation of CXCR4 and CXCR7 expression at the promoter level in RF/6A cells.

In vivo study, we found that intravitreous injection of the LPS increased the size of experimental CNV, accompanying with the up-regulation of CXCR4, CXCR7, SDF-1 and IL-6 expression. The size of CNV was suppressed by anti-SDF-1 antibody in LPS-treated rat. IL-6, a potent proinflammatory cytokine, has been proved to be a risk factor for CNV generation in previous studies [36]. In the current study, the concentration of IL-6 was also up-regulated after LPS treatment, which further confirmed the effect of LPS on CNV. However, we didn’t found a significant difference in IL-10 level between LPS-treated and control groups. A recent study by Matsumura et al. [37] reported that CNV formation was suppressed by low-dose LPS pretreatment via IL-10 production by macrophages. This conflict may explanation for the different administration route (vitreous injection vs. intraperitoneal injection) of LPS. Further studies will be required to elucidate the effect of LPS with different doses and administration route in laser-induced CNV model.

In summary, activation of the LPS-TLR4 pathway in endothelial cells gave them the abilities of migration and tube formation via the up-regulation of CXCR4 and CXCR7 levels. In vivo experiments validated the effectiveness of SDF-1 neutralization for CNV treatment. These data could help us to understand better the mechanism of action of the TLR4 signaling pathway, as well as of the CXCR7/CXCR4/SDF-1 axis, in CNV formation, thus adding new insights to the development of more effective therapies for the treatment of CNV.

## Supporting Information

S1 Fig Effects of LPS on activation of the MAPK and NF-κB signaling pathways in RF/6A cells. (A) RF/6A cells were treated with LPS (1 μg/ml) for 5, 15, 30, 60 and 120 min, and the phosphorylation of JNK, p38-MAPK and ERK1/2 was detected by western blot. LPS activated ERK1/2 and JNK in a time-dependent manner, as evidenced by the increases in phosphorylated ERK1/2 and JNK, but not p-38. (B) Chemical inhibitors against MEK1/2 (U0126; 10 μM) and JNK (SP600125; 10 μM) inhibited increases in ERK1/2 and JNK phosphorylation, respectively. (C) The cytosol and nuclear protein of LPS-treated RF/6A were separated and analyzed by western blotting for NF-κB translocation. (D) Immunofluorescence microscopic analysis was performed for the identification of NF-κB location 24 h after LPS treatment. NF-κB was labeled by green fluorescence (FITC), and the nuclei were stained by blue fluorescence (DAPI). (E) NF-κB transcriptional activity was assayed by co-transfection with NF-κB-dependent and Renilla-dependent luciferase reporter plasmids for 24 h, followed by stimulation with LPS (1 μg/ml) alone or LPS and BAY11-7082 for another 24 h. Data are shown as the mean ± SD of four separate experiments. Statistical significance determined using one-way ANOVA with *P < 0.05, **P < 0.01.
(TIF)

S2 Fig LPS treatment increased SDF-1 concentration, CXCR4 and CXCR7 expression in the eye. (A) Total RNA of retina-RPE-choroid complex was extracted, and the amounts of CXCR4 and CXCR7 (stimulated for 24 h) mRNA were quantified by qRT-PCR and normalized to the corresponding amounts of GAPDH mRNA. (B) Eye tissues (cornea, iris, vitreous body, retina, choroids, and sclera) were homogenized and the supernatants were subjected to ELISA, and the concentration of SDF-1, IL-6 and IL-10 (stimulated for 24 h) was measured. Data shown are mean ± SD of triplicate samples and are representative of four independent experiments. Statistical significance determined using Student’s *t* test with *P < 0.05, **P < 0.01.
(TIF)
