# Supplementary figures and images for: Lipopolysaccharide Promotes Choroidal Neovascularization by Up-Regulation of CXCR4 and CXCR7 Expression in Choroid Endothelial Cell
Source: PLoS One. 2015 Aug 19;10(8):e0136175. doi: 10.1371/journal.pone.0136175 (PMC4545586; doi:10.1371/journal.pone.0136175)

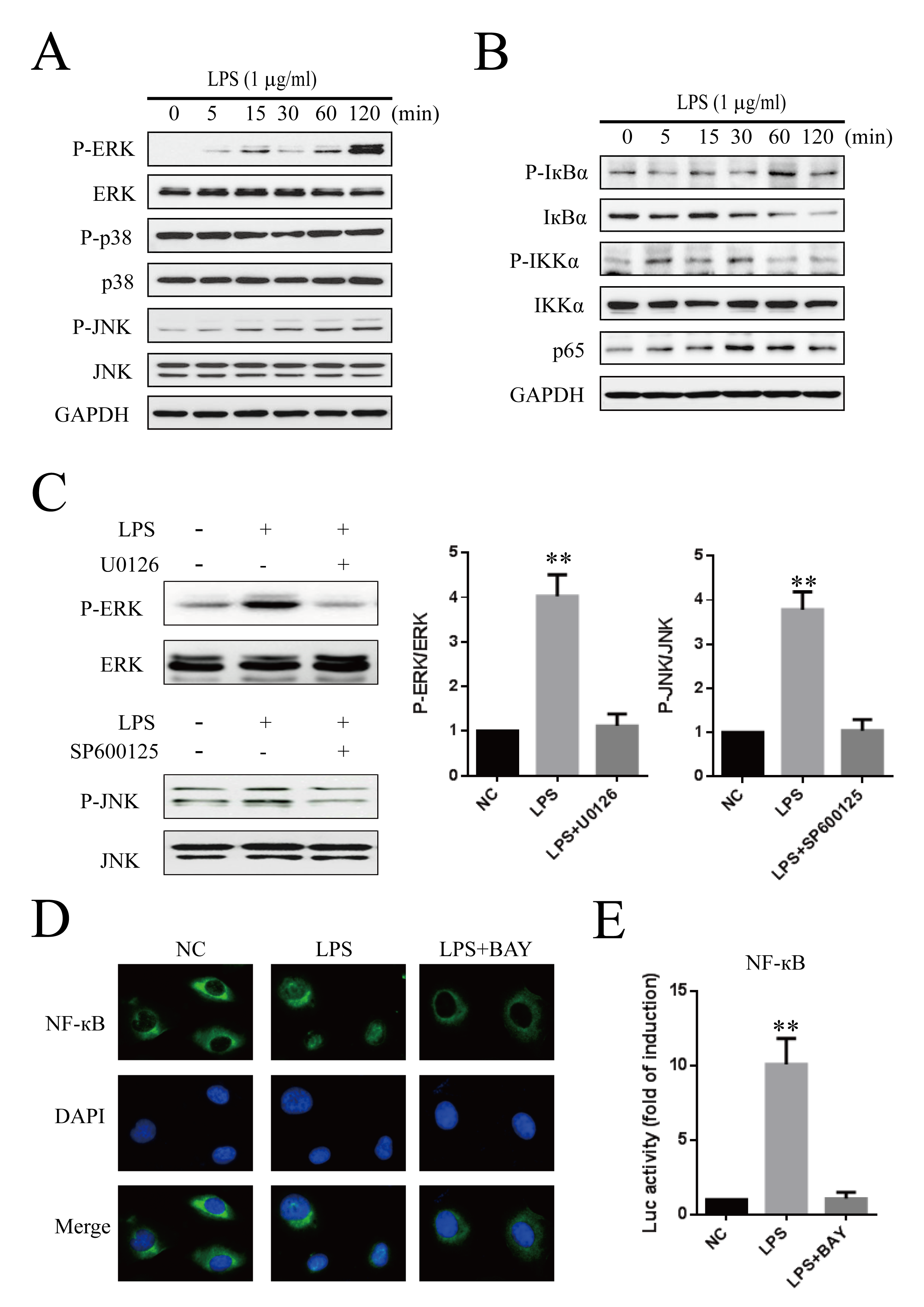

Supplement: S1 Fig — (A) RF/6A cells were treated with LPS (1 μg/ml) for 5, 15, 30, 60 and 120 min, and the phosphorylation of JNK, p38-MAPK and ERK1/2 was detected by western blot. LPS activated ERK1/2 and JNK in a time-dependent manner, as evidenced by the increases in phosphorylated ERK1/2 and JNK, but not p-38. (B) Chemical inhibitors against MEK1/2 (U0126; 10 μM) and JNK (SP600125; 10 μM) inhibited increases in ERK1/2 and JNK phosphorylation, respectively. (C) The cytosol and nuclear protein of LPS-treated RF/6A were separated and analyzed by western blotting for NF-κB translocation. (D) Immunofluorescence microscopic analysis was performed for the identification of NF-κB location 24 h after LPS treatment. NF-κB was labeled by green fluorescence (FITC), and the nuclei were stained by blue fluorescence (DAPI). (E) NF-κB transcriptional activity was assayed by co-transfection with NF-κB-dependent and Renilla-dependent luciferase reporter plasmids for 24 h, followed by stimulation with LPS (1 μg/ml) alone or LPS and BAY11-7082 for another 24 h. Data are shown as the mean ± SD of four separate experiments. Statistical significance determined using one-way ANOVA with *P < 0.05, **P < 0.01. (TIF) [file pone.0136175.s001.tif]

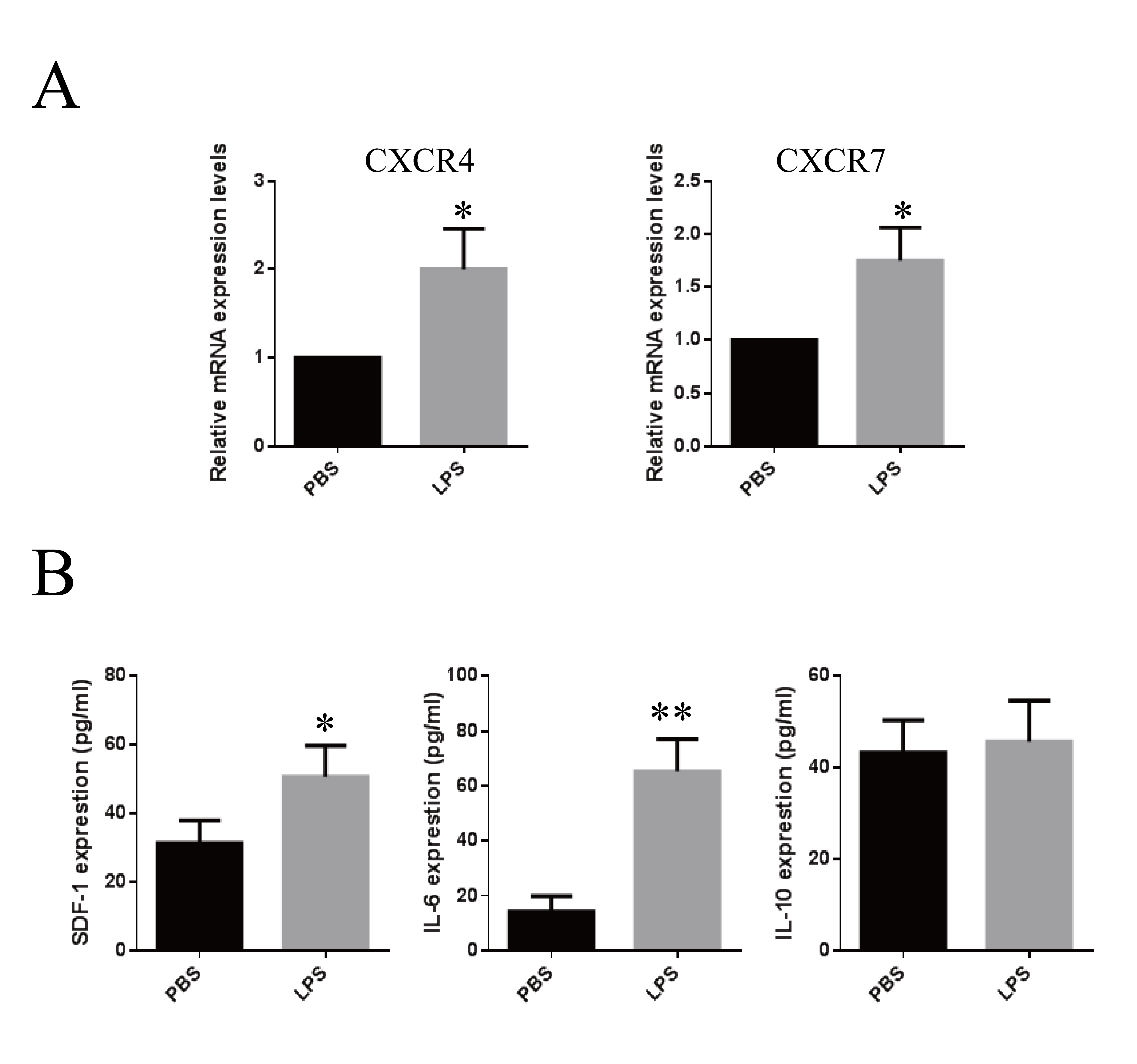

Supplement: S2 Fig — (A) Total RNA of retina-RPE-choroid complex was extracted, and the amounts of CXCR4 and CXCR7 (stimulated for 24 h) mRNA were quantified by qRT-PCR and normalized to the corresponding amounts of GAPDH mRNA. (B) Eye tissues (cornea, iris, vitreous body, retina, choroids, and sclera) were homogenized and the supernatants were subjected to ELISA, and the concentration of SDF-1, IL-6 and IL-10 (stimulated for 24 h) was measured. Data shown are mean ± SD of triplicate samples and are representative of four independent experiments. Statistical significance determined using Student’s t test with *P < 0.05, **P < 0.01. (TIF) [file pone.0136175.s002.tif]
